# A comparison of the efficacy and safety of combined aclidinium bromide and formoterol fumarate in the treatment of chronic obstructive pulmonary disease: A protocol for systematic review and meta-analysis

**DOI:** 10.1097/MD.0000000000021856

**Published:** 2020-11-20

**Authors:** Hong Lu, Yi-Tong Huang, He-Jiang Chen, Pei-Feng Chen

**Affiliations:** Department of Respiratory, Zhuji Affiliated Hospital of Shaoxing University, Zhuji, Zhejiang, P.R. China.

**Keywords:** aclidinium bromide, chronic obstructive pulmonary disease, efficacy, formoterol fumarate, safety

## Abstract

**Background:**

To systematically evaluate the efficacy and safety of combined aclidinium bromide and formoterol fumarate for chronic obstructive pulmonary disease (COPD).

**Methods:**

Electronic databases including PubMed, MEDLINE, EMBASE, Cochrane Library Central Register of Controlled Trials, WanFang, and China National Knowledge Infrastructure (CNKI) database were searched for studies on the use of combined aclidinium bromide and formoterol fumarate in the treatment of COPD. Two independent researchers performed literature screening, data extraction, and assessment of quality of studies. The strength of the association of the efficacy and safety of combined aclidinium bromide and formoterol fumarate in the treatment of COPD was evaluated according to the odds ratio (OR), mean differences (MDs), and 95% confidence interval (CI). Statistical analysis was carried out via using RevMan 5.3 software.

**Results:**

The results of the present study will be published in a peer-reviewed journal.

**Conclusion:**

The conclusion of the present study will provide evidence to judge whether combined aclidinium bromide and formoterol fumarate is an effective and safety intervention in the treatment of COPD.

**Systematic review registration number:**

INPLASY202070063.

## 1 Introduction

Chronic obstructive pulmonary disease (COPD) is characterized by a persistent airflow limitation that is usually progressive and associated with an enhanced chronic inflammatory response in the airways.^[1,2]^ It is the third leading cause of death in the world, with 328 million in the global estimated to have COPD in 2010.^[3,4]^ Symptoms of COPD are multifaceted and the most common symptoms include wheeze, long-term cough, sputum production, breathlessness. For the treatment of COPD patients, inhaled drugs, including aclidinium bromide, play a vital role in reducing the exacerbation risk and symptoms.^[5]^ However, many patients still suffered from dyspnea and substantial limitations in daily life. Thus, it is necessary to seek effective and safety intervention in the treatment of COPD.

Clinical and experimental studies have confirmed that combined aclidinium bromide and formoterol fumarate can reduce the severity of inflammation, improve the lung function, improve the quality of life, and reduce the frequency of acute exacerbations.^[6–8]^ However, no systematic review has been performed to evaluate the efficacy and safety of combined aclidinium bromide and formoterol fumarate for COPD. Accordingly, this systematic review will evaluate the efficacy and safety of combined aclidinium bromide and formoterol fumarate in the treatment of COPD.

## 2 Materials and methods

This study has been registered at International Platform of Registered Systematic Review and Meta-analysis Protocols (INPLASY, https://inplasy.com/). The registration DOI number of the present study is 10.37766/inplasy2020.7.0063. In addition, this review protocol has been reported in line with PRISMA (Preferred Reporting Items for Systematic Review and Meta-Analyses) guidelines.^[9]^

## 3 Eligibility criteria

### 3.1 Types of studies

All the included studies were double-blind, randomized, parallel-group studies, evaluating combined aclidinium bromide and formoterol fumarate as fixed-dose combination (FDC) in the treatment of COPD. We excluded any other studies, such as observational study, non-randomized control studies, case report.

### 3.2 Types of participants

We included adults (over 18 years old) with a diagnosis of COPD based on the criteria of the Global Initiative for Chronic Obstructive Lung Disease,^[10]^ the World Health Organization,^[11]^ or Chinese Medical Association Respiratory Disease Society for COPD guideline.^[12]^ Participants with COPD will be included regardless of their age, sex, region, or other factors. We excluded studies that enrolled participants with other respiratory system diseases, such as bronchial asthma, cystic fibrosis or other chronic lung diseases.

### 3.3 Types of interventions and comparisons

We included trials comparing combined aclidinium bromide and formoterol fumarate as fixed-dose combination (FDC) versus aclidinium bromide, formoterol fumarate. Aclidinium bromide and formoterol fumarate must have been part of the randomly assigned treatments in the included studies. There will be no limitation on the duration of trials.

### 3.4 Types of outcome measures

#### 3.4.1 Primary outcomes

Lung function, including postbronchodilator forced expiratory volume in 1 second (FEV_1_), forced vital capacity (FVC), and FEV_1_/FVC.

#### 3.4.2 Secondary outcomes

(i) Exacerbations (number of participants experiencing one or more exacerbations) requiring a short course of an oral steroid or antibiotic, or both.
(ii) Quality of life as measured by St George's Respiratory Questionnaire (SGRQ) score change from the baseline.
(iii) Improvement in symptoms as measured by the Transitional Dyspnoea Index (TDI) change from the baseline.
(iv) Adverse events, including serious adverse events (SAEs), not including SAEs, and total adverse events.

### 3.5 Search methods

#### 3.5.1 Electronic searches

Electronic databases including PubMed, MEDLINE, EMBASE, Cochrane Library Central Register of Controlled Trials, WanFang, and China National Knowledge Infrastructure (CNKI) database were searched for studies on the use of combined aclidinium bromide and formoterol fumarate in the treatment of COPD. All the above databases will be searched from inception to the present. No language and publication time restriction will be used.

#### 3.5.2 Searching other resources

We searched ClinicalTrials.gov (www.ClinicalTrials.gov), Google scholar, and reference lists of review articles and all primary studies for additional studies.

#### 3.5.3 Search strategy

The search strategy is as follows: (“chronic obstructive pulmonary disease” OR “chronic obstructive lung disease” OR “chronic obstructive respiratory disease” OR “chronic obstructive airway disease” OR “COPD”) AND (“aclidinium bromide” OR “formoterol fumarate” OR “aclidinium bromide and formoterol fumarate”) AND (“randomized clinical trial” OR “randomized controlled trial” OR “randomized” OR “RCT”).

### 3.6 Data collection and analysis

#### 3.6.1 Selection of studies

**Figure 1:**
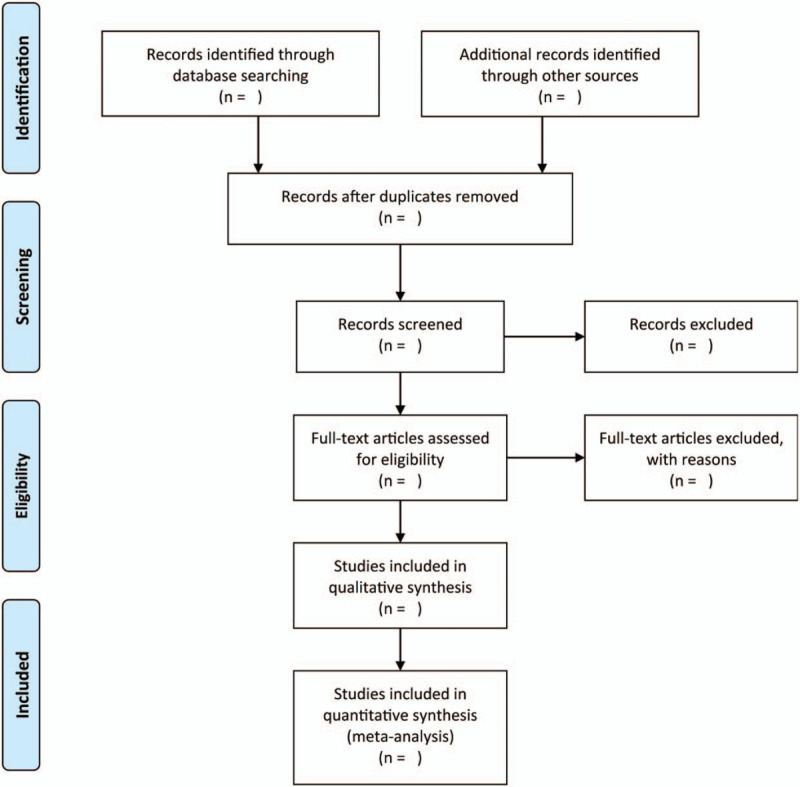
Flow diagram of the literature search.

Two independent researchers (HYT and CPF) performed literature selection based on the previously designed eligibility criteria. Any disagreement will be resolve by discussion, or, if required, we consulted the third (LH) author. Titles and abstracts of searched literatures will be identified to exclude duplicates and any irrelevant studies. The process of study selection will be shown in the PRISMA flow chart (Fig. 1).

#### 3.6.2 Data extraction

For all studies included, at least 2 authors (CPF and CHJ) will extract relevant information independently, and then import data into Excel tables. Information will include author, publication year, the criteria of diagnostic, eligibility criteria, details of treatment and control interventions, duration of intervention, lung function, and outcome indicators. Any disagreement will be resolve by discussion, or, if required, we consulted the third author (LH).

#### 3.6.3 Assessment of study quality

The 2 authors evaluate the quality of the included studies independently using the criteria outlined in the Cochrane Collaboration's tool.^[13]^ Any disagreements between the 2 authors will be resolved by discussion or by consultation with another author. We assessed the risk of bias of each included study by using following domains: selection bias, detection bias, reporting bias, performance bias, attrition bias, and other source of bias. We grade each potential source of bias as 3 levels: “High risk,” “Low risk,” or “Unclear risk.”

#### 3.6.4 Measures of treatment effect

We analyses dichotomous variables by using odds ratios (ORs) and continuous variables by using mean difference (MD) with its 95% confidence intervals (CIs) using RevMan 5.3 (Cochrane, London, UK).

#### 3.6.5 Assessment of heterogeneity

The statistical heterogeneity among included studies will be detected using standard Chi-squared statistic and *I*^*2*^ test^[14]^: *I*^*2*^ < 50% or *P*-value > .1 means minor heterogeneity, the fixed-effects model will be used to pooled the data^[15]^; while *I*^*2*^ > 50% or *P*-value < .1 suggests considerable heterogeneity, the random-effects model will be used to pooled the data.^[16]^

#### 3.6.6 Assessment of reporting biases

The funnel plot and Egger test will be applied to evaluate the potential publication bias when >10 studies are included.^[17,18]^

#### 3.6.7 Sensitivity analysis

Sensitivity analysis will be applied to test the stability and robustness of study finding via exclusion of studies with high-risk of bias or unclear methodological data.

#### 3.6.8 Subgroup analysis

We planned to carry out the following subgroup analyses: Dosage of LABAs (e.g., formoterol fumarate 6 μg, formoterol fumarate 12 μg). We also planned to use the following outcomes in subgroup analyses: exacerbations requiring a short course of an oral steroid or antibiotic, or both. Quality of life. Adverse events.

### 3.7 Ethics and dissemination

Ethical approval will not be required for the present systematic review. In the present study, patients are not recruited and data are not collected from patients. This review will be disseminated via peer-reviewed journal.

## 4 Discussion

Although published studies have reported that combined aclidinium bromide and formoterol fumarate have been used for the treatment of COPD,^[6–8]^ but the results are still controversial. In addition, no systematic review has been performed to evaluate the efficacy and safety of combined aclidinium bromide and formoterol fumarate for COPD. Accordingly, this systematic review will evaluate the efficacy and safety of combined aclidinium bromide and formoterol fumarate in the treatment of COPD. The findings of the present study may provide evidence for clinicians and health-related professionals to make clinical decisions to improve COPD treatment approach.

## Author contributions

**Data curation:** Hong Lu, He-Jiang Chen, and Pei-Feng Chen.

**Formal analysis:** Hong Lu, He-Jiang Chen.

**Investigation:** Pei-Feng Chen.

**Methodology:** Hong Lu.

**Resources:** Pei-Feng Chen.

**Software:** Yi-Tong Huang, He-Jiang Chen.

**Supervision:** Yi-Tong Huang.

**Visualization:** He-Jiang Chen.

**Writing:** Hong Lu and Yi-Tong Huang.
